# Long non‐coding RNA HCG11 sponging miR‐522‐3p inhibits the tumorigenesis of non‐small cell lung cancer by upregulating SOCS5

**DOI:** 10.1111/1759-7714.13624

**Published:** 2020-08-26

**Authors:** Gang Fan, Jin Jiao, Feng Shen, Qingxia Ren, Qing Wang, Fulu Chu

**Affiliations:** ^1^ Department of Clinical Laboratory Shandong Provincial Hospital Affiliated to Shandong First Medical University Jinan China; ^2^ Department of Clinical Laboratory Shandong Maternal and Child Health Care Hospital Jinan China; ^3^ Department of Clinical Laboratory People's Hospital of Rizhao Rizhao China; ^4^ Department of Imaging The People's Hospital of Zhangqiu Area Jinan China

**Keywords:** HCG11, miR‐522‐3p, non‐small cell lung cancer, SOCS5

## Abstract

**Background:**

Numerous studies have shown that long non‐coding RNA (lncRNA) is involved in various human diseases including non‐small cell lung cancer (NSCLC). The aim of this study was to explore the potential role of lncRNA HCG11 in the pathogenesis of NSCLC.

**Methods:**

The mRNA expression of HCG11, miR‐522‐3p and SOCS5 was detected by RT‐qPCR. The regulatory mechanism of lncRNA HCG11 was investigated by CCK‐8, transwell and dual luciferase reporter assays.

**Results:**

Downregulation of lncRNA HCG11 and upregulation of miR‐522‐3p were found in NSCLC tissues and cells, and abnormal expressions of lncRNA HCG11 and miR‐522‐3p were related to adverse clinical outcomes of NSCLC patients. LncRNA HCG11 acted as a molecular sponge for miR‐522‐3p. Functionally, lncRNA HCG11 inhibited cell viability, migration and invasion in NSCLC by downregulating miR‐522‐3p. Further, miR‐522‐3p directly targeted SOCS5. lncRNA HCG11 could positively regulate SOCS5 expression in NSCLC. In addition, HCG11 downregulation or miR‐522‐3p overexpression abolished the inhibitory effect of SOCS5 on cell viability, migration and invasion in NSCLC.

**Conclusions:**

LncRNA HCG11 inhibits cell viability, migration and invasion in NSCLC by functioning as a ceRNA of miR‐522‐3p to upregulate SOCS5.

## Graphical abstract

Downregulation of lncRNA HCG11 is related to adverse clinical outcomes in NSCLC patients. HCG11 has been shown to inhibit cell viability and motility in NSCLC, and HCG11 functions as a ceRNA of miR‐522‐3p to upregulate SOCS5.

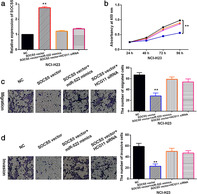

## Introduction

Non‐small cell lung cancer (NSCLC) is one of the most common cancers that cause cancer death, accounting for 85%–90% of lung cancer.^1^, ^2^ NSCLC includes squamous cell carcinoma, adenocarcinoma, and large cell carcinoma. Compared with small cell carcinoma, NSCLC cells grow and divide slowly, and metastasize relatively late.^3^ Previous studies have shown that smoking, occupational and environmental exposure, ionizing radiation, and genetics are associated with NSCLC occurrence.^4^ At present, the treatment of NSCLC mainly includes surgery, radiotherapy, chemotherapy, molecular targeting and immunotherapy.^5^ However, the prognosis for patients with NSCLC is poor. About 75% of NSCLC patients are found to be in the middle and late stage of disease, and the five‐year survival rate is reported to be very low.^6^ Moreover, the timely detection and treatment of early lung cancer can significantly improve a patient's prognosis. Therefore, exploring effective biomarkers for the early diagnosis of NSCLC is important.

Long non‐coding RNAs (lncRNAs) have become a hot spot in the field of non‐coding RNA research.^7^ Current research suggests that the functional mechanism of lncRNA mainly includes the regulation of transcription, post‐transcriptional regulation, protein transport and mRNA degradation.^8^, ^9^ Many studies have shown that lncRNAs can act as either tumor suppressors or promoters in the occurrence and development of NSCLC, such as PCNA‐AS1 and FTX.^10^, ^11^ LncRNA HLA complex group 11 (HCG11) has been reported to be involved in human cancers. For example, it has been previously reported that lncRNA HCG11 accelerated the progression of hepatocellular carcinoma via miR‐26a‐5p/ATG12 axis,^12^ and lncRNA HCG11 promoted cell proliferation and migration in gastric cancer via targeting miR‐1276/CTNNB1.^13^ However, lncRNA HCG11 suppressed laryngeal carcinoma cell progression via sponging the miR‐4469/APOM axis.^14^ LncRNA HCG11 has been found to inhibit glioma progression through cooperating with miR‐496/CPEB3 axis.^15^ These studies indicate that the function of lncRNA HCG11 is tissue specific. Moreover, the role of lncRNA HCG11 in NSCLC remains unclear.

Mechanistically, lncRNAs can exert function in human cancers by acting as competing endogenous RNAs (ceRNA) to regulate microRNA/gene axis. Here, miR‐522‐3p was predicted to have a binding site with lncRNA HCG11. More importantly, downregulation of miR‐522 has been found to suppress proliferation and metastasis of NSCLC cells by directly targeting DENND2D.^16^ In this study, suppressor of cytokine signaling 5 (SOCS5) was found to be a potential target of miR‐522‐3p. The role of SOCS5 has been investigated in human cancers. Downregulation of SOCS5 has been detected in prostate and liver cancers.^17^, ^18^ Functionally, lncRNA FER1L4 has been reported to induce apoptosis and suppress EMT in osteosarcoma cells via inhibiting miR‐18a‐5p to promote SOCS5.^19^ However, the function of SOCS5 as well as its relationship with lncRNA HCG11 or miR‐522‐3p has not been reported in previous studies.

Therefore, this study was designed to elucidate the role of lncRNA HCG11 and regulatory mechanism of HCG11/miR‐522‐3p/SOCS5 in NSCLC. These results may potentiate the discovery of new therapies for NSCLC.

## Methods

### Clinical tissues

A total of 62 NSCLC tissues and their adjacent normal tissues were collected from patients in People's Hospital of Rizhao. Informed consents were obtained from all NSCLC patients. None of the patients had received radiotherapy or chemotherapy before surgery. This research was approved by the Institutional Ethics Committee of People's Hospital of Rizhao. The tissues were frozen in liquid nitrogen and stored at −80°C.

### Cell culture and transfection

Human normal bronchial epithelial cell line BEAS‐2B and lung cells NCI‐H23 were purchased from the American Type Culture Collection (ATCC, Manassas, VA, USA). The growth conditions were 5% CO_2_, 37°C and culture solution (90% DMEM medium +10% FBS).

HCG11 or SOCS5 overexpression plasmid was obtained by cloning HCG11 or SOCS5 fragment into pcDNA3.1 empty vector (Invitrogen, Carlsbad, CA, USA). Short hairpin RNA (shRNA) against HCG11, miR‐522‐3p mimics or inhibitor was synthesized by GenePharma (Suzhou, China). The above‐mentioned plasmids and oligonucleotides were transfected into NCI‐H23 cells by using Lipofectamine 2000 reagents (Invitrogen). Untreated NCI‐H23 cells were used as the control (NC).

### RT‐qPCR

Total RNA was extracted from tissues and cells using TRIZOL reagent (Invitrogen, USA) and the isolated RNA was treated with RNase‐free DNase I (Promega Corporation, Madison, WI, USA). The concentration and purity of isolated RNA were measured with a spectrophotometer. Purified RNA (0.5 μg/μL) with nuclease‐free water was used for cDNA synthesis using PrimeScript RT Reagent Kit (TaKaRa, Dalian, China). RT‐qPCR assay was performing using SYBR Premix Ex Taq (TaKaRa, Dalian, China) based on the manufacturer's instruction. PCR was conducted under the following parameters: one predenaturation cycle of one minute at 94°C, 34 cycles of 95°C for 15 seconds, 59°C for 30 seconds, 72°C for two minutes and a final extension at 72°C for five minutes. The mRNA and miRNA levels were normalized by GAPDH or U6. The 2^–ΔΔCt^ method was used to calculate the expressions of HCG11, miR‐522‐3p and SOCS5. The primers used were: HCG11 forward 5′‐GCT CTA TGC CAT CCT GCT T‐3′; reverse 5′‐TCC CAT CTC CAT CAA CCC‐3′; miR‐522‐3p forward: 5′‐ACA CTC CAG CTG GGC TCT AGA GGG AAG CGC‐3′ and reverse, 5′‐TGG TGT CGT GGA GTC G‐3′; U6‐forward: 5′‐GCT TCG GCA GCA CAT ATA CTA AAA T‐3′ and reverse, 5′‐CGC TTC ACG AAT TTG CGT GTC AT‐3′; SOCS5 forward: 5′‐ATA AGT GGA GAT GGT TCT GC‐3′ and reverse, 5′‐TCC TCC TGT GCA GAG TCC‐3′; GAPDH forward: 5′‐ACA ACT TTG GTA TCG TGG AAG G‐3′, and reverse, 5′‐GCC ATC ACG CCA CAG TTT C‐3′.

### Cell counting kit‐8 (CCK‐8) assay

Transfected NCI‐H23 cells (4 × 10^3^ cells/well) in 96‐well plates were incubated for 24, 48, 72 or 96 hours, respectively. The cells were then incubated with 10 μL CCK‐8 reagents for four hours. The medium was discarded and dimethyl sulfoxide was added. The absorbance at 450 nm was detected by a microplate reader (Olympus Corp., Tokyo, Japan).

### Transwell assay

First, 60 μL of diluted Matrigel (3.9 μg/μL) was added to the upper chamber for cell invasion. The cell migration assay was performed without Matrigel. After 30 minutes, NCI‐H23 cell suspension (3 × 10^3^ cells/well) was added to the transwell upper chamber, and 500 μL of DMEM medium (10% FBS) was added to 24‐well plates in the lower chamber. After 24 hours, 0.1% crystal violet was applied to stain the moved cells. The stained cells were observed with a microscope (Nikon, Tokyo, Japan) in five randomly chosen fields.

### Dual luciferase reporter assay

The wild‐type 3′‐UTR of HCG11 or SOCS5 containing the speculated binding sites for miR‐522‐3p was cloned into the downstream of psiCHECK‐2 vector (Promega, Fitchburg, WI, USA). The mutant HCG11 or SOCS5 luciferase reporter vector was then constructed by mutating the miR‐522‐3p binding sites. NCI‐H23 cells were cotransfected with miR‐522‐3p mimics and the luciferase vectors using Lipofectamine 2000 (Invitrogen; Thermo Fisher Scientific, Inc.) according to the manufacturer's protocol. Next, the luciferase activity was observed by dual‐luciferase reporter assay system (Promega, USA) 48 hours after cell transfection. At 48 hours post‐transfection, the relative reporter activity was normalized by Renilla luciferase activity.

### Statistical analysis

Data were analyzed using SPSS 17.0 or Graphpad Prism 6 and shown as mean ± SD. Chi‐squared test was used to analyze the association between the clinical features and lncRNA HCG11 or miR‐522‐3p. Differences were analyzed using Student *t*‐test and one‐way analysis of variance with Tukey's post hoc test. Statistical significance was set at *P* < 0.05.

## Results

### LncRNA HCG11 and miR‐522‐3p are abnormally expressed in NSCLC

**Figure 1:**
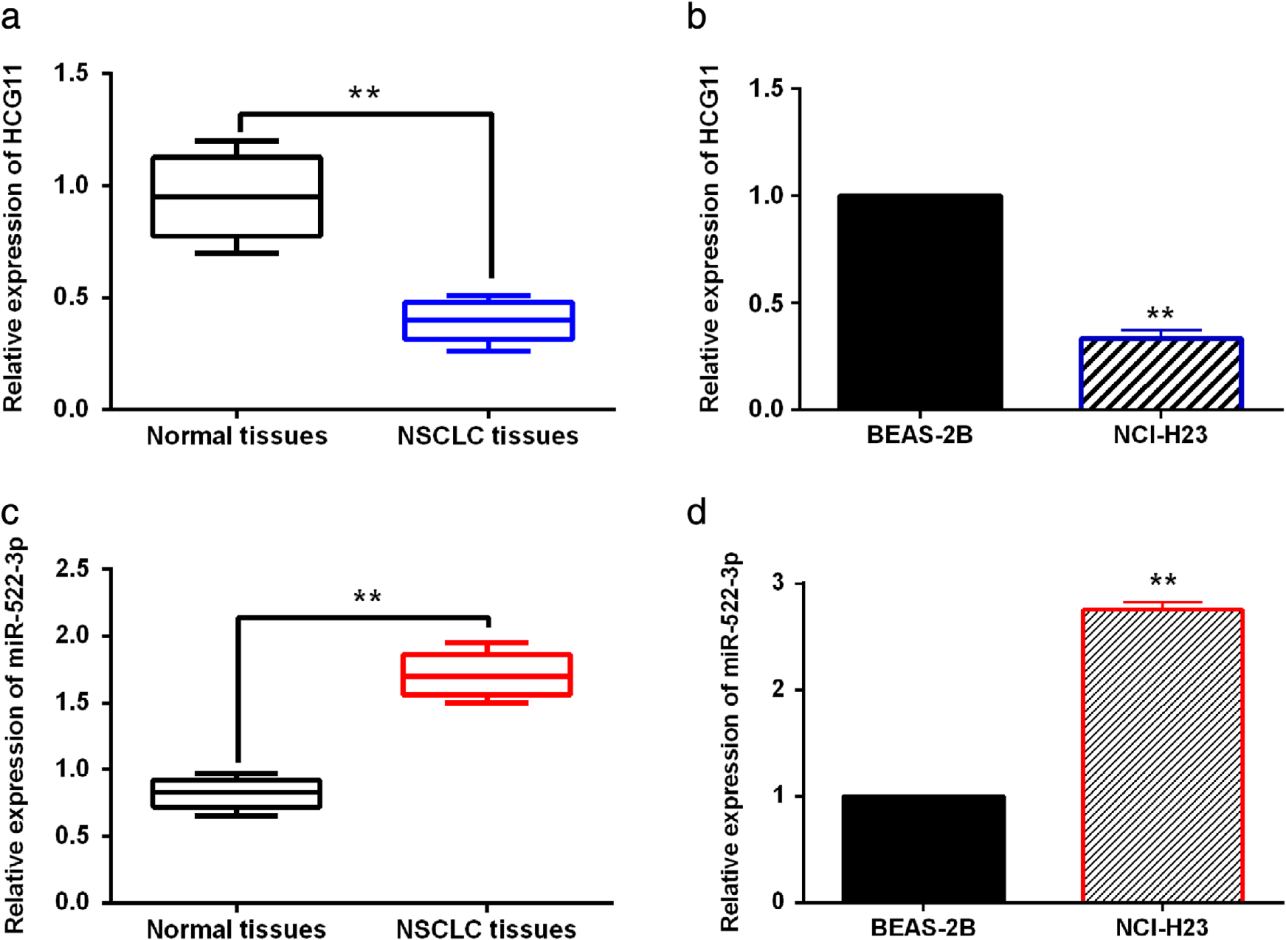
LncRNA HCG11 and miR‐522‐3p are abnormally expressed in NSCLC. (**a**) LncRNA HCG11 expression was detected in NSCLC tissues and normal tissues. (**b**) LncRNA HCG11 expression was compared between NCI‐H23 NSCLC cells and human normal bronchial epithelial cell line BEAS‐2B. (**c**) MiR‐522‐3p expression was compared between NSCLC tissues and normal tissues. (**d**) MiR‐522‐3p expression was detected in NCI‐H23 NSCLC cells and human normal bronchial epithelial cell line BEAS‐2B. **P < 0.01.

First, the expression levels of lncRNA HCG11 and miR‐522‐3p was examined in NSCLC tissues and cells. RT‐qPCR showed that lncRNA HCG11 expression in NSCLC tissues was lower than that in normal tissues (Fig 1a). Similarly, downregulation of lncRNA HCG11 was detected in NCI‐H23 NSCLC cells compared to human normal bronchial epithelial cell line BEAS‐2B (Fig 1b). Moreover, abnormal expression of lncRNA HCG11 was related to tumor size, TNM stage and lymph nodes metastasis in NSCLC patients (Table 1). Next, we found that miR‐522‐3p was upregulated in NSCLC tissues compared to normal tissues (Fig 1c). Compared to human normal bronchial epithelial cell line BEAS‐2B, miR‐522‐3p expression was increased in NSCLC cells NCI‐H23 (Fig 1d). In addition, abnormal expression of miR‐522‐3p was associated with TNM stage and lymph nodes metastasis in NSCLC patients (Table 1). These results indicate that lncRNA HCG11 and miR‐522‐3p may be involved in the pathogenesis of NSCLC.

**Table 1:** Relationship between lncRNA HCG11 or miR‐522‐3p expression and clinicopathological characteristics in NSCLC patients

		HCG11			miR‐522‐3p		
Characteristics	Cases	High	Low	*P*‐value	High	Low	*P*‐value
Age (years)				0.29			0.38
≥60	40	12	28		22	18	
<60	22	5	17		16	6	
Gender				0.31			0.33
Male	42	9	33		28	14	
Female	20	8	12		10	10	
Tumor size (mm)				0.04*			0.07
≤3	45	11	34		29	16	
>3	17	6	11		9	8	
Lymph node metastasis				0.04*			0.03*
Yes	22	7	15		9	13	
No	40	10	30		29	11	
TNM stage				0.03*			0.03*
I–II	41	9	32		26	15	
III–IV	21	8	13		12	9	

Statistical analyses were performed by the χ^2^ test.

* *P* < 0.05 was considered statistically significant.

### LncRNA HCG11 acts as a molecular sponge for miR‐522‐3p

**Figure 2:**
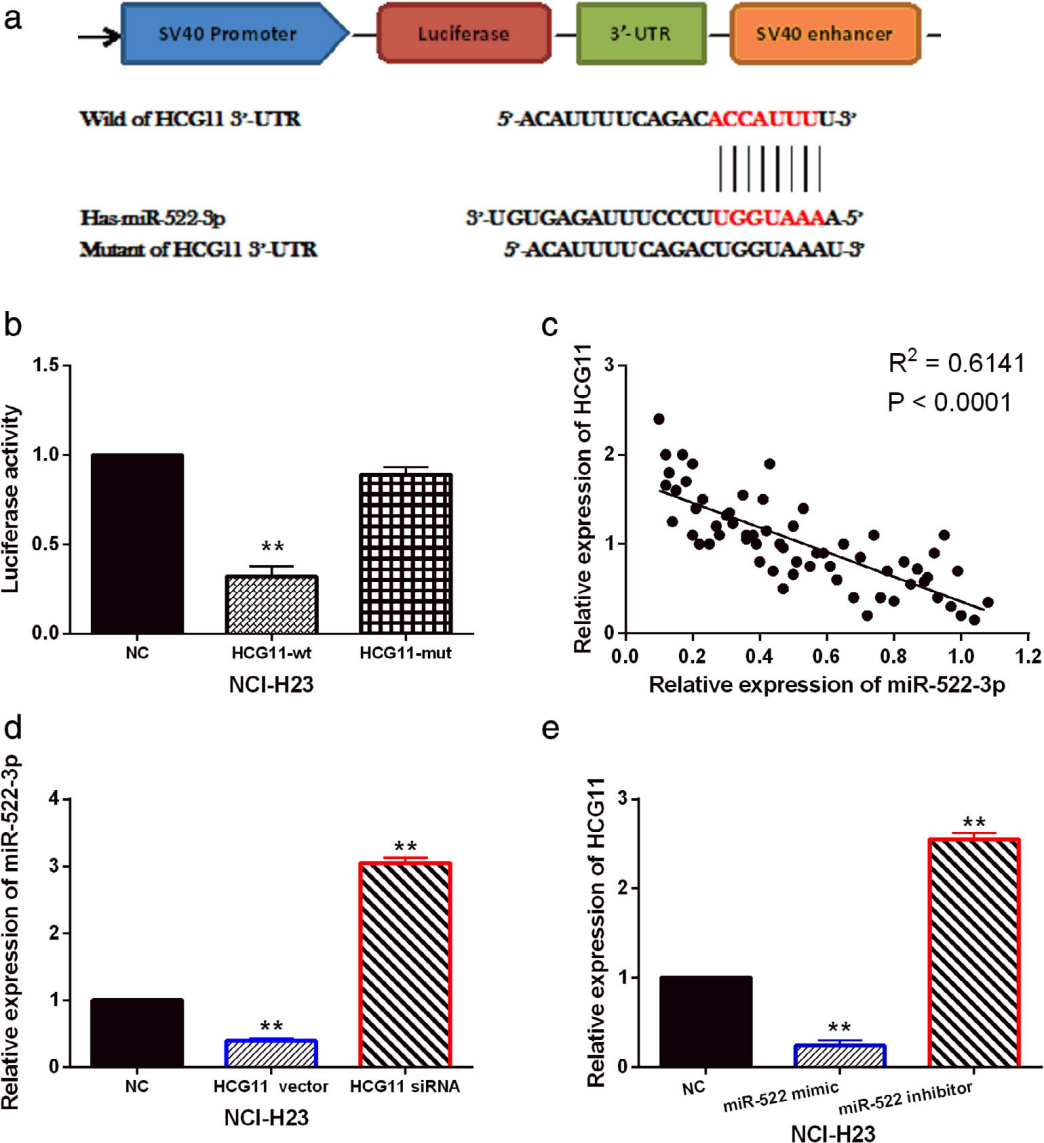
LncRNA HCG11 acts as a molecular sponge for miR‐522‐3p. (**a**) The binding sites between HCG11 with miR‐522‐3p. (**b**) Luciferase reporter assay was performed to confirm the relationship between HCG11 with miR‐522‐3p. (**c**) MiR‐522‐3p was negatively correlated with HCG11 expression in NSCLC tissues. (**d**) MiR‐522‐3p expression was detected in NCI‐H23 cells with HCG11 siRNA and vector. (**e**) HCG11 expression was detected in NCI‐H23 cells containing miR‐522‐3p mimics or inhibitor. Untreated NCI‐H23 cells were used as the control (NC). **P < 0.01.

The starBase database (http://starbase.sysu.edu.cn/) predicts that lncRNA HCG11 has a binding site with miR‐522‐3p (Fig 2a). Dual luciferase reporter assay was designed to verify the above prediction. The results showed that miR‐522‐3p mimics reduced the luciferase activity of wt‐HCG11, but had little effect on mut‐HCG11 in NCI‐H23 cells (Fig 2b). Moreover, lncRNA HCG11 was negatively correlated with miR‐522‐3p expression in NSCLC tissues (Fig 2c). Next, we found that HCG11 vector reduced miR‐522‐3p expression, while HCG11 siRNA inhibited miR‐522‐3p expression in NCI‐H23 cells (Fig 2d). Meanwhile, lncRNA HCG11 expression was reduced by miR‐522‐3p mimics and promoted by miR‐522‐3p inhibitor in NCI‐H23 cells (Fig 2e). These findings indicate that lncRNA HCG11 acts as a molecular sponge for miR‐522‐3p. A negative correlation between lncRNA HCG11 and miR‐522‐3p expression was identified in NSCLC tissues and cells.

### LncRNA HCG11 inhibits cell viability and motility in NSCLC by downregulating miR‐522‐3p

**Figure 3:**
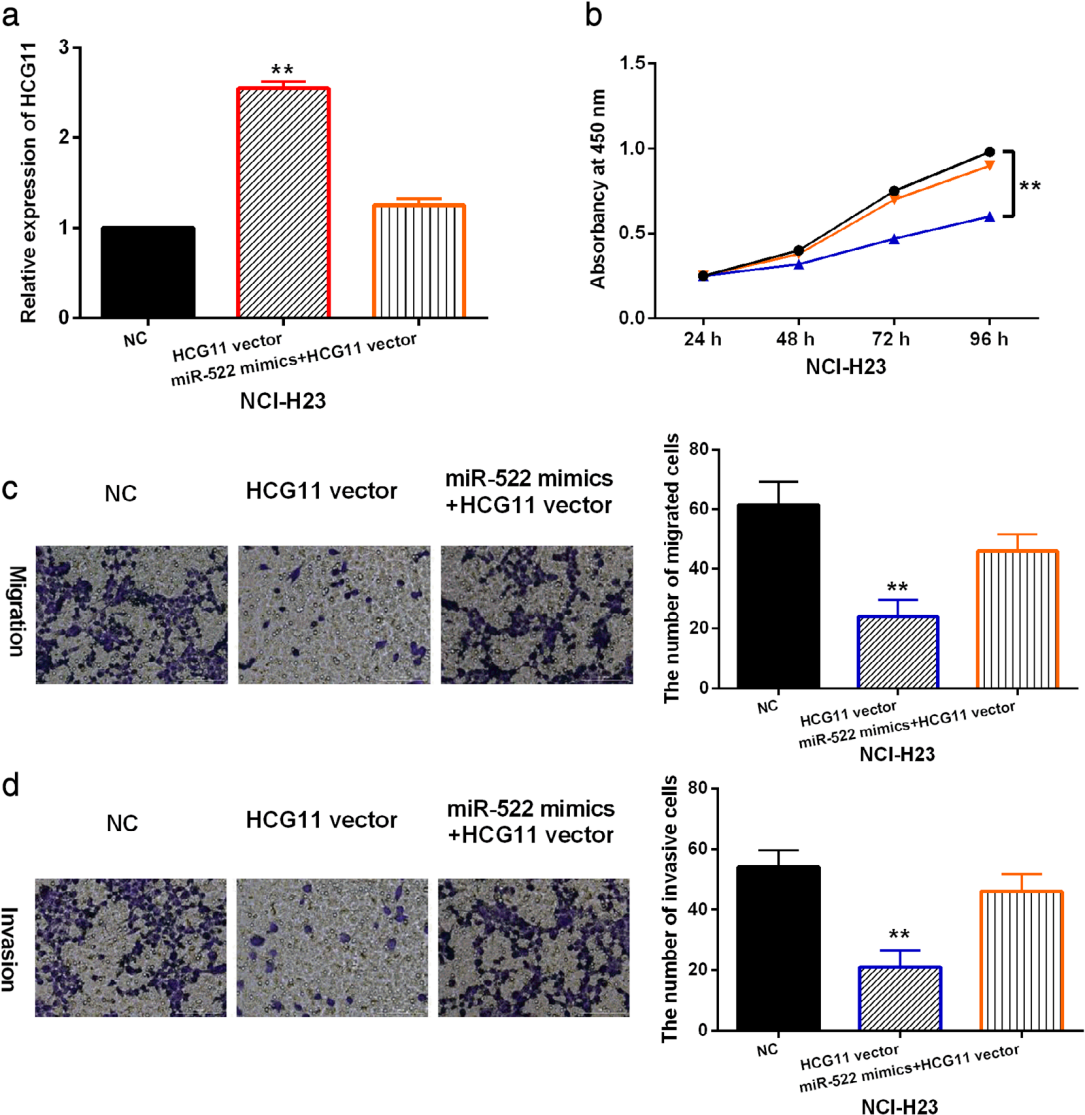
LncRNA HCG11 inhibits cell viability and motility in NSCLC by downregulating miR‐522‐3p. (**a**) LncRNA HCG11 expression was detected in NCI‐H23 cells with HCG11 vector or HCG11 vector + miR‐522 mimics. (**b**, **c**, **d**) Cell proliferation, migration and invasion were detected in NCI‐H23 cells with HCG11 vector or HCG11 vector + miR‐522 mimics. Untreated NCI‐H23 cells were used as the control (NC). **P < 0.01. 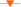 miR‐522 mimics + HCG11 vector, 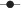 NC, 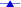 HCG11 vector.

Next, the functional mechanism of lncRNA HCG11/miR‐522‐3p was investigated in NSCLC cells. HCG11 vector or HCG11 vector+miR‐522‐3p mimics was transfected into NCI‐H23 cells. RT‐qPCR showed that HCG11 expression was enhanced by its vector in NCI‐H23 cells. After transfection of miR‐522‐3p mimics, the increased expression of HCG11 was reduced in NCI‐H23 cells (Fig 3a). CCK‐8 assay indicated that overexpression of HCG11 inhibited cell proliferation in NCI‐H23 cells, and miR‐522‐3p overexpression abolished the inhibitory effect of HCG11 on cell proliferation in NCI‐H23 cells (Fig 3b). Transwell assay suggested that overexpression of HCG11 inhibited NCI‐H23 cell migration and invasion, and the inhibitory effect of HCG11 on NCI‐H23 cell migration and invasion was also weakened by miR‐522‐3p overexpression (Fig 3c,d). Taken together, lncRNA HCG11 inhibited cell viability and motility in NSCLC by downregulating miR‐522‐3p.

### SOCS5 is a direct target of miR‐522‐3p

**Figure 4:**
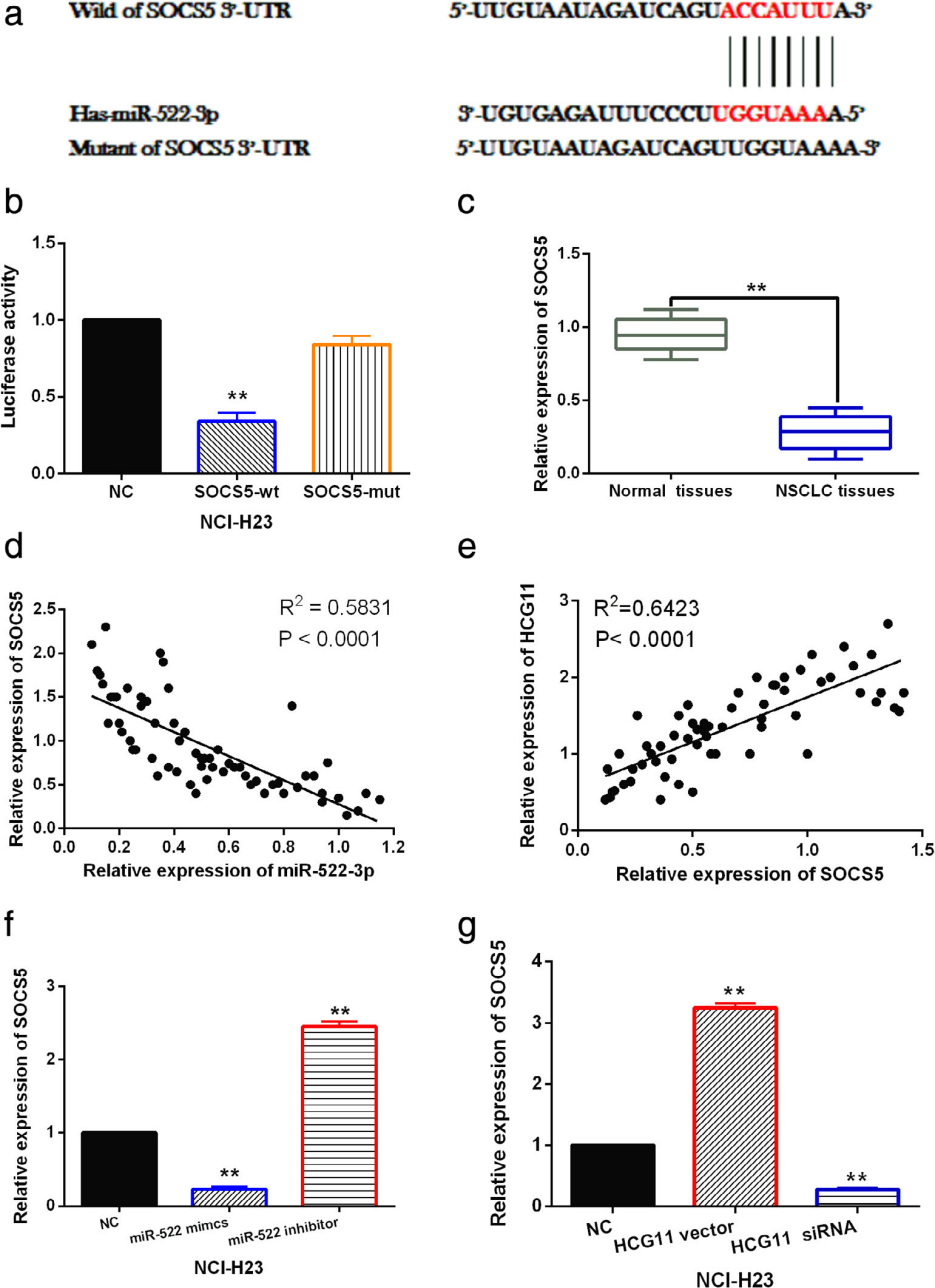
SOCS5 is a direct target of miR‐522‐3p. (**a**) The binding site between SOCS5 and miR‐522‐3p. (**b**) Luciferase reporter assay was performed to confirm the relationship between SOCS5 with miR‐522‐3p. (**c**) SOCS5 expression was compared between NSCLC tissues and normal tissues. (**c**) MiR‐522‐3p was negatively correlated with SOCS5 expression in NSCLC tissues. (**d**) SOCS5 was positively correlated with HCG11 expression in NSCLC tissues. (**e**) SOCS5 expression was detected in NCI‐H23 cells with miR‐522‐3p mimics or inhibitor. (**f**) SOCS5 expression was detected in NCI‐H23 cells with HCG11 siRNA or vector. Untreated NCI‐H23 cells were used as the control (NC). **P < 0.01.

Further, TargetScan database (http://www.targetscan.org) predicts that miR‐522‐3p has a binding site with the 3'‐UTR of SOCS5 (Fig 4a). Moreover, the role of SOCS5 has been widely investigated in human cancers (exclude NSCLC), indicating that SOCS5 can be stably expressed in human cells and is involved in tumorigenesis. Luciferase reporter assay showed that miR‐522‐3p mimics reduced the luciferase activity of wt‐SOCS5, but did not affect mut‐SOCS5 luciferase activity (Fig 4b). It demonstrates that miR‐522‐3p directly targets SOCS5. Meanwhile, downregulation of SOCS5 was found in NSCLC tissues compared to normal tissues (Fig 4c). Furthermore, miR‐522‐3p was negatively correlated with SOCS5 expression in NSCLC tissues (Fig 4d), and a positive correlation between HCG11 and SOCS5 expression was found in NSCLC tissues (Fig 4e). In addition, miR‐522‐3p mimics reduced SOCS5 expression, while miR‐522‐3p inhibitor promoted SOCS5 expression in NCI‐H23 cells (Fig 4f). However, SOCS5 expression was enhanced by HCG11 overexpression and decreased by HCG11 downregulation in NCI‐H23 cells (Fig 4g). Collectively, we determined that miR‐522‐3p directly targeted SOCS5, and lncRNA HCG11 can positively regulate SOCS5 expression in NSCLC.

### LncRNA HCG11/miR‐522‐3p inhibits NSCLC tumorigenesis by regulating SOCS5

**Figure 5:**
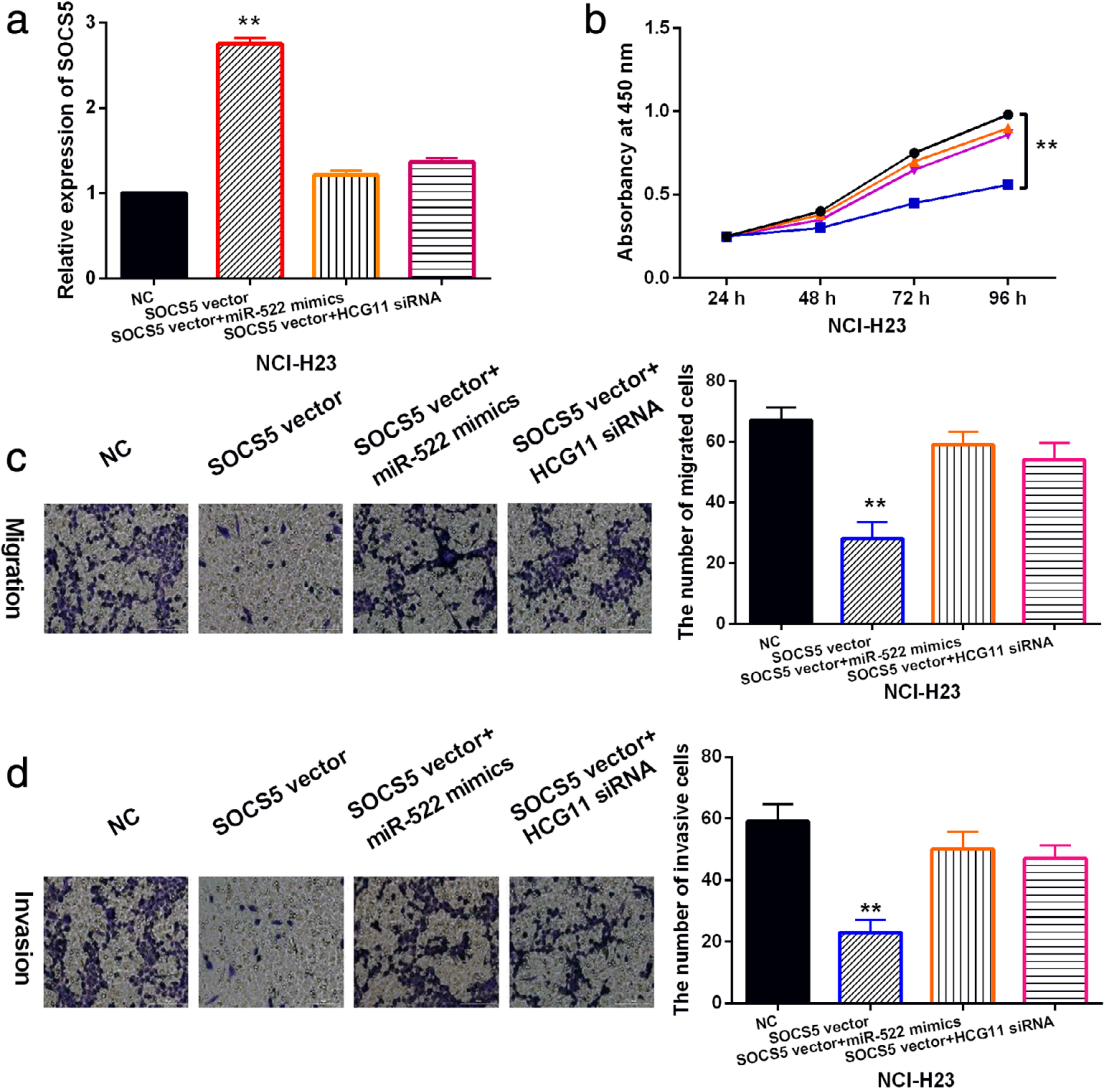
LncRNA HCG11/miR‐522‐3p inhibits NSCLC tumorigenesis by regulating SOCS5. (**a**) SOCS5 expression was detected in NCI‐H23 cells with SOCS5 vector, SOCS5 vector + miR‐522 mimics or SOCS5 vector + HCG11 siRNA. (**b**, **c**, **d**) Cell proliferation, migration and invasion were detected in NCI‐H23 cells with SOCS5 vector, SOCS5 vector + miR‐522 mimics or SOCS5 vector + HCG11 siRNA. Untreated NCI‐H23 cells were used as the control (NC). **P < 0.01. 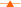 SOCS5 vector + miR‐522 mimics, 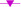 SOCS5 vector + HCG11 siRNA, 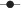 NC, 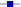 SOCS5 vector

To investigate whether SOCS5 regulates the tumorigenesis of NSCLC by participating in lncRNA HCG11/miR‐522‐3p axis, HCG11 siRNA or miR‐522‐3p mimics was transfected into NCI‐H23 cells containing SOCS5 vector. RT‐qPCR showed that the increased expression of SOCS5 induced by SOCS5 vector was restored by HCG11 siRNA or miR‐522‐3p mimics (Fig 5a). Functionally, upregulation of SOCS5 was found to inhibit NCI‐H23 cell proliferation. However, HCG11 siRNA or miR‐522‐3p mimics weakened the inhibitory effect of SOCS5 on cell proliferation in NCI‐H23 cells (Fig 5b). Additionally, cell migration and invasion were restrained by SOCS5 overexpression in NCI‐H23 cells, and HCG11 downregulation or miR‐522‐3p overexpression abolished the inhibitory effect of SOCS5 on NCI‐H23 cell migration and invasion (Fig 5c,d). Collectively, we determined that lncRNA HCG11 inhibited the tumorigenesis of NSCLC by sponging miR‐522‐3p and upregulating SOCS5.

## Discussion

Recently, increasing numbers of lncRNAs have been reported to be aberrantly expressed and participate in the tumorigenesis of NSCLC, such as CCAT1 and SNHG6.^20^, ^21^ In this study, downregulation of lncRNA HCG11 was found in NSCLC and was related to adverse clinical outcomes of NSCLC patients. Consistent with our results, decreased expression of lncRNA HCG11 has been identified in cervical cancer and hepatocellular carcinoma.^22^, ^23^ In addition, downregulation of lncRNA HCG11 has been reported to be associated with poor clinical outcomes and prognosis in patients with prostate cancer,^24^ which is similar to our results. Functionally, overexpression of HCG11 has been found to inhibit cell proliferation, invasion and migration, whereas induce cell apoptosis in prostate cancer.^25^ Here, we also found that upregulation of lncRNA HCG11 inhibited cell viability, migration and invasion in NSCLC. These findings indicated that lncRNA HCG11 acts as tumor suppressor in the development of NSCLC.

Since the “competing endogenous RNA” (ceRNA) hypothesis was proposed in 2011,^26^ the sponging effect of lncRNAs on microRNAs has attracted more and more attention. Similarly, lncRNA HCG11 has been reported to suppress the growth of glioma through cooperating with miR‐4425/MTA3 axis.^27^ In the present study, lncRNA HCG11 acted as a molecular sponge for miR‐522‐3p. Upregulation of miR‐522‐3p has also been detected in NSCLC and is associated with adverse clinical outcomes in NSCLC patients. Zhang *et al*. also reported that miR‐522 expression was increased in NSCLC, and downregulation of miR‐522 suppressed proliferation and metastasis of NSCLC cells.^16^ Moreover, we found that lncRNA HCG11 inhibited cell viability and motility in NSCLC by downregulating miR‐522‐3p. Similarly, overexpression of LINC00261 has also been found to inhibit NSCLC cell progression by interacting with miR‐522‐3p.^28^ These results indicate that miR‐522‐3p functions as a tumor promoter in NSCLC.

In addition, we found that SOCS5 was a downstream target of miR‐522‐3p. And SOCS5 was downregulated in NSCLC tissues. The expression of SOCS5 was negatively correlated with miR‐522‐3p level but positively correlated with lncRNA HCG11 level in NSCLC. Functionally, upregulation of SOCS5 was found to inhibit cell proliferation, migration and invasion in NSCLC. Consistent with our study, downregulation and inhibitory effect of SOCS5 have been found in breast and pancreatic cancer.^29^, ^30^ In the current study, overexpression of miR‐522‐3p was found to weaken the inhibitory effect of SOCS5 in NSCLC, suggesting that miR‐522‐3p promotes the development of NSCLC by downregulating SOCS5. Su *et al*. also reported that miR‐885‐5p upregulation promoted colorectal cancer cell proliferation and migration by targeting suppressor of SOCS5.^31^ Additionally, lncRNA HAND2‐AS1 has been reported to inhibit liver cancer cell proliferation and migration by upregulating SOCS5.^32^ Here, downregulation of HCG11 weakened the inhibitory effect of SOCS5 in NSCLC, indicating that lncRNA HCG11 restrains NSCLC progression by upregulating SOCS5.

In conclusion, this study demonstrated that lncRNA HCG11 expression was decreased in NSCLC tissues and cells, and abnormal expression of lncRNA HCG11 was related to adverse clinical outcomes in NSCLC patients. Functionally, lncRNA HCG11 inhibited cell viability, migration and invasion in NSCLC by functioning as a ceRNA of miR‐522‐3p to upregulate SOCS5. Therefore, our study indicates that lncRNA HCG11 may be a potential therapy target for NSCLC.

## Disclosure

The authors declare that they have no competing interests.

## References

[1] Li MM , Wang X , Yun ZY , Wang RT , Yu KJ . Platelet indices in non‐small cell lung cancer patients with brain metastases. Cancer Biomark 2019; 24: 515–9. 30932885 10.3233/CBM-192393 PMC13082525

[2] Sun D , Cao M , Li H , He S , Chen W . Cancer burden and trends in China: A review and comparison with Japan and South Korea. Chin J Cancer Res 2020; 32: 129–39. 32410791 10.21147/j.issn.1000-9604.2020.02.01 PMC7219092

[3] Torre LA , Bray F , Siegel RL , Ferlay J , Lortet‐Tieulent J , Jemal A . Global cancer statistics, 2012. CA Cancer J Clin 2015; 65: 87–108. 25651787 10.3322/caac.21262

[4] Singh N , Behera D . Lung cancer epidemiology and clinical profile in North India: Similarities and differences with other geographical regions of India. Indian J Cancer 2013; 50: 291. 24369196 10.4103/0019-509X.123581

[5] Ballatori E , Fatigoni S , Roila F . Treatment of lung cancer. New Eng J Med 2009; 361: 2485; author reply 2486‐2487. 10.1056/NEJMc0909634 20018971

[6] Zappa C , Mousa SA . Non‐small cell lung cancer: Current treatment and future advances. Trans Lung Cancer Res 2016; 5: 288–300. 10.21037/tlcr.2016.06.07 PMC4931124 27413711

[7] Lu T , Wang Y , Chen D , Liu J , Jiao W . Potential clinical application of lncRNAs in non‐small cell lung cancer. Onco Targets Ther 2018; 11: 8045–52. 30519046 10.2147/OTT.S178431 PMC6239124

[8] Yan X , Hu Z , Feng Y *et al* Comprehensive genomic characterization of long non‐coding RNAs across human cancers. Cancer Cell 2015; 28: 529–40. 26461095 10.1016/j.ccell.2015.09.006 PMC4777353

[9] Ginn L , Shi L , Montagna M , Garofalo M . LncRNAs in non‐small‐cell lung cancer. Non‐cod RNA 2020; 6: 25. 10.3390/ncrna6030025 PMC7549371 32629922

[10] Wu C , Zhu XT , Xia L *et al* High expression of long noncoding RNA PCNA‐AS1 promotes non‐small‐cell lung cancer cell proliferation and oncogenic activity via upregulating CCND1. J Cancer 2020; 11: 1959–67. 32194807 10.7150/jca.39087 PMC7052854

[11] Jin S , He J , Zhou Y , Wu D , Li J , Gao W . LncRNA FTX activates FOXA2 expression to inhibit non‐small‐cell lung cancer proliferation and metastasis. J Cell Mol Med 2020; 24: 4839–49. 32176463 10.1111/jcmm.15163 PMC7176842

[12] Li ML , Zhang Y , Ma LT . LncRNA HCG11 accelerates the progression of hepatocellular carcinoma via miR‐26a‐5p/ATG12 axis. Eur Rev Med Pharmacol Sci 2019; 23: 10708–20. 31858580 10.26355/eurrev_201912_19771

[13] Zhang H , Huang H , Xu X *et al* LncRNA HCG11 promotes proliferation and migration in gastric cancer via targeting miR‐1276/CTNNB1 and activating Wnt signaling pathway. Cancer Cell Int 2019; 19: 350. 31889902 10.1186/s12935-019-1046-0 PMC6933929

[14] Xue HX , Li HF , Wang T , Li WJ , Bian WC . LncRNA HCG11 suppresses laryngeal carcinoma cells progression via sponging miR‐4469/APOM axis. Eur Rev Med Pharmacol Sci 2020; 24: 3174–82. 32271435 10.26355/eurrev_202003_20684

[15] Chen Y , Bao C , Zhang X , Lin X , Huang H , Wang Z . Long non‐coding RNA HCG11 modulates glioma progression through cooperating with miR‐496/CPEB3 axis. Cell Prolif 2019; 52: e12615. 31310044 10.1111/cpr.12615 PMC6797506

[16] Zhang T , Hu Y , Ju J *et al* Downregulation of miR‐522 suppresses proliferation and metastasis of non‐small cell lung cancer cells by directly targeting DENN/MADD domain containing 2D. Sci Rep 2016; 6: 19346. 26783084 10.1038/srep19346 PMC4726064

[17] Seashols‐Williams SJ , Budd W , Clark GC *et al* miR‐9 acts as an OncomiR in prostate cancer through multiple pathways that drive tumour progression and metastasis. PLoS One 2016; 11: e0159601. 27447934 10.1371/journal.pone.0159601 PMC4957825

[18] Sanchez‐Mejias A , Kwon J , Chew XH *et al* A novel SOCS5/miR‐18/miR‐25 axis promotes tumorigenesis in liver cancer. Int J Cancer 2019; 144: 311–21. 30191950 10.1002/ijc.31857

[19] Ye F , Tian L , Zhou Q , Feng D . LncRNA FER1L4 induces apoptosis and suppresses EMT and the activation of PI3K/AKT pathway in osteosarcoma cells via inhibiting miR‐18a‐5p to promote SOCS5. Gene 2019; 721: 144093. 31473323 10.1016/j.gene.2019.144093

[20] Li N , Hao W , Yang J , Guo Y , Guo Y , Du Y . Long non‐coding RNA colon cancer‐associated transcript‐1 regulates tumor cell proliferation and invasion of non‐small‐cell lung cancer through suppressing miR‐152. Geriatr Gerontol Int 2020; 20: 629–36. 32227563 10.1111/ggi.13914

[21] Li K , Jiang Y , Xiang X *et al* Long non‐coding RNA SNHG6 promotes the growth and invasion of non‐small cell lung cancer by downregulating miR‐101‐3p. Thoracic Cancer 2020; 11: 1180–90. 32147945 10.1111/1759-7714.13371 PMC7180593

[22] Zhang Y , Zhang X , Zhu H *et al* Identification of potential prognostic long non‐coding RNA biomarkers for predicting recurrence in patients with cervical cancer. Cancer Manag Res 2020; 12: 719–30. 32099468 10.2147/CMAR.S231796 PMC7002755

[23] Xu Y , Zheng Y , Liu H , Li T . Modulation of IGF2BP1 by long non‐coding RNA HCG11 suppresses apoptosis of hepatocellular carcinoma cells via MAPK signaling transduction. Int J Oncol 2017; 51: 791–800. 28677801 10.3892/ijo.2017.4066 PMC5564403

[24] Zhang Y , Zhang P , Wan X *et al* Downregulation of long non‐coding RNA HCG11 predicts a poor prognosis in prostate cancer. Biomed Pharmacother 2016; 83: 936–41. 27522256 10.1016/j.biopha.2016.08.013

[25] Wang YC , He WY , Dong CH , Pei L , Ma YL . lncRNA HCG11 regulates cell progression by targeting miR‐543 and regulating AKT/mTOR pathway in prostate cancer. Cell Biol Int 2019; 43: 1453–62. 10.1002/cbin.11194 31228307

[26] Salmena L , Poliseno L , Tay Y , Kats L , Pandolfi PP . A ceRNA hypothesis: The Rosetta stone of a hidden RNA language? Cell 2011; 146: 353–8. 21802130 10.1016/j.cell.2011.07.014 PMC3235919

[27] Zhang L , Cao Y , Kou X *et al* Long non‐coding RNA HCG11 suppresses the growth of glioma by cooperating with the miR‐4425/MTA3 axis. J Gene Med 2019; 21: e3074. 30706982 10.1002/jgm.3074

[28] Shi J , Ma H , Wang H *et al* Overexpression of LINC00261 inhibits non‐small cell lung cancer cells progression by interacting with miR‐522‐3p and suppressing Wnt signaling. J Cell Biochem 2019; 120: 18378–87. 31190356 10.1002/jcb.29149

[29] Liu C , Li W , Zhang L , Song C , Yu H . Tumor‐suppressor microRNA‐151‐5p regulates the growth, migration and invasion of human breast cancer cells by inhibiting SCOS5. Am J Trans Res 2019; 11: 7376–84. PMC6943465 31934285

[30] Hu H , Zhang Q , Chen W *et al* MicoRNA‐301a promotes pancreatic cancer invasion and metastasis through the JAK/STAT3 signaling pathway by targeting SOCS5. Carcinogenesis 2020; 41: 502–14. 31233116 10.1093/carcin/bgz121

[31] Su M , Qin B , Liu F , Chen Y , Zhang R . miR‐885‐5p upregulation promotes colorectal cancer cell proliferation and migration by targeting suppressor of cytokine signaling. Oncol Lett 2018; 16: 65–72. 29928388 10.3892/ol.2018.8645 PMC6006474

[32] Yan D , Jin F , Lin Y . lncRNA HAND2‐AS1 inhibits liver cancer cell proliferation and migration by upregulating SOCS5 to inactivate the JAK‐STAT pathway. Cancer Biother Radiopharm 2020; 35: 143–52. 32155348 10.1089/cbr.2019.2958

